# Interactive Errors Analysis and Scale Factor Nonlinearity Reduction Methods for Lissajous Frequency Modulated MEMS Gyroscope

**DOI:** 10.3390/s23249701

**Published:** 2023-12-08

**Authors:** Rui Li, Xiaoxu Wang, Kaichen Yan, Zhennan Chen, Zhengya Ma, Xiquan Wang, Ao Zhang, Qianbo Lu

**Affiliations:** 1 Institute of Flexible Electronics, Northwestern Polytechnical University, 127 West Youyi Road, Beilin District, Xi’an 710072, China; nwpu_lirui@mail.nwpu.edn.cn; 2 School of Automation, Northwestern Polytechnical University, 127 West Youyi Road, Beilin District, Xi’an 710072, China

**Keywords:** Lissajous frequency modulation (LFM), scale factor nonlinearity, stiffness coupling, phase error, frequency difference

## Abstract

Although the Lissajous frequency modulated (LFM) mode can improve the long-term and temperature stability of the scale factor (SF) for mode mismatch MEMS gyroscopes, its SF nonlinearity poses a significant limitation for full-scale accuracy maintenance. This paper examines the interaction effects among stiffness coupling, system phase delay, readout demodulation phase shift, and velocity amplitude mismatch within the control process. Based on the completion of frequency difference control and demodulation phase matching, we clarify that the remaining stiffness coupling and residual system phase error are the primary factors influencing SF nonlinearity. Furthermore, SF nonlinearity is reduced through error compensation. On one hand, this paper suppresses stiffness coupling through the observation of the instantaneous frequency difference and the application of the quadrature voltage. On the other hand, system phase error is compensated by observing the amplitude control force and tuning the reference in the Phase-Locked Loops (PLLs). Subsequent simulations of these methods demonstrated a remarkable 97% reduction in SF nonlinearity within the measurement range of ±500°/s. In addition, an observed rule dictates that maintaining a sufficiently large frequency split effectively constrains the SF nonlinearity.

## 1. Introduction

The MEMS gyroscope has been widely used in consumer electronics, autonomous driving, industry, and even near-inertial navigation [1,2]. However, conventional amplitude modulated (AM) mode gyroscopes face challenges in improving the SF stability, temperature stability, and dynamic range [3,4,5,6]. Recently, frequency modulated (FM) mode operations, like the quadrature FM (QFM) mode, indexed FM (IFM) mode, and fully differential FM mode, have emerged as potential alternatives due to their superior temperature stability, wider dynamic range, and increased measurement bandwidth [7,8,9,10,11]. Unfortunately, the aforementioned FM modes necessitate mode matching, which involves complex operations including mechanical trimming, stiffness tuning, and mode-matching control [2]. In contrast, the LFM operation only necessitates equal amplitude control and natural frequency maintenance, significantly simplifying the implementation process [12,13]. By leveraging the principle of continue-time mode reversal, the LFM mode enhances the gyroscope performance without mode matching, resulting in advantages such as a more stable SF, reduced sensitivity to temperature variations, a higher quality factor, and lower power consumption. Furthermore, while the anisodamping error leads to angle-dependent drift in the whole angle (WA) mode, it is effectively eliminated in the LFM mode with the aid of differential operations [14]. Coincidentally, mass-produced fully symmetric MEMS gyroscopes (e.g., the micro-hemispherical resonant gyroscope, disk resonant gyroscope, etc.) typically exhibit high-quality factors and significant frequency splits with the current production process [15,16]. Implementing the LFM operation on them would improve their performance without complex tuning. LFM gyroscopes can be widely used in tactical applications, offering benefits in terms of cost, size, weight, and power (CSWaP).

In the preceding decade, considerable advancements have been achieved on LFM gyroscopes, specifically pertaining to the structural design, circuitry construction, and electromechanical integration. In the domain of structural design, the utilization of quad-mass resonators as the operational units for the LFM has emerged, complementing the conventional lumped mass MEMS resonators [12]. Notably, a dual-mass pitch gyroscope with an out-of-plane structure has been innovatively designed, alongside the development of resonators supporting three-axis detection [17,18]. Regarding circuit construction, a specialized sigma–delta frequency-to-digital conversion circuit has been instrumental in enhancing the frequency detection resolution significantly [19]. Additionally, a circuit promoting real-time phase extraction and digital demodulation has been devised, effectively achieving phase-matching readout [20]. Furthermore, the introduction of an all-digital control circuit based on the digital PLL has been proposed, eliminating the need for specialized frequency readout circuits [21]. Concerning electromechanical integration, the inception of the fully digital output Application-Specific Integrated Circuit (ASIC) has laid a robust foundation for LFM commercialization [22]. These works aim to augment the fundamental capabilities of the LFM and propel its product applications. Discussions of the LFM performance usually revolve around reducing random noise, with research endeavors targeting enhancements in the frequency stability, demodulator accuracy, and comprehensive noise analysis within the system. Nevertheless, as a gyroscope with a substantial measurement range, investigations into the SF nonlinearity of the LFM are rarely present in the research.

**Figure 1:**
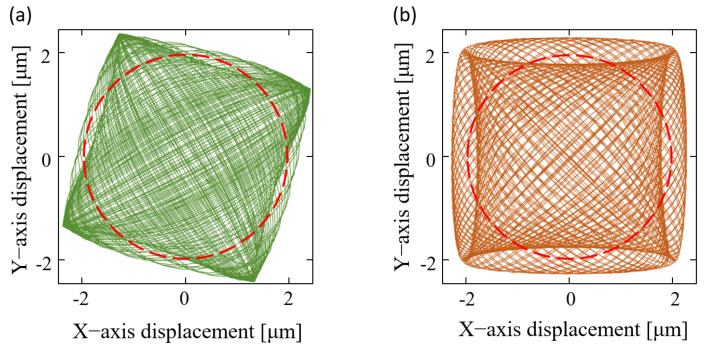
Patterns of distorted Lissajous trajectories: (**a**) The green trajectory is the pattern with a 1.15% velocity amplitude mismatch caused by a 500 dsp angular rate. (**b**) The orange trajectory is the pattern with stiffness coupling caused by the 20 Hz frequency split and 5 deg stiffness axis deflection. The red circle with a displacement of 2 μm in the center is the circular trajectory generated by the standard pattern.

SF nonlinearity serves as a critical indicator of gyroscope stability, correlating with the measurement range and requiring careful consideration [23]. It is caused by errors within the system, including stiffness coupling, system phase error, readout module phase shift, and velocity amplitude mismatch. Among these errors, the issue of phase shift, as a significant concern in circuit research, has already been addressed through various approaches. The velocity amplitude mismatch is the primary factor influencing the SF, as illustrated by the distorted Lissajous trajectory in Figure 1a. It is dependent on the stabilization of the frequency difference and amplitude, which varies with the input angular rate [24]. On the one hand, although utilizing electrostatic force for frequency difference control has been investigated, this necessitates an additional electrostatic voltage to the driving electrode, resulting in increased power consumption [25]. On the other hand, AGC-less control methods and control methods with separate differential and integral operations can suppress fluctuations in amplitude [26,27]. Furthermore, advanced control methods for MEMS gyroscopes can greatly enhance the control effectiveness. These methods include the data-driven control scheme, the nonsingular terminal sliding mode control method, an approximation-based adaptive fractional sliding mode control scheme, and so on [28,29,30]. Hence, research on stiffness coupling and system phase error is crucial for further constraining the SF nonlinearity. The stiffness coupling between the two detection modes results in a significant quadrature error and leads to the generation of distorted Lissajous trajectories, as depicted in Figure 1b. While the AM mode mitigates the stiffness coupling through quadrature tuning during mode matching, no relevant aspects of the LFM model have been reported [31]. The phase lags exist in each block of the MEMS system, contributing to driving force deflection and resulting in PLL tracking errors. While the impact of phase errors has been studied in AM and WA operations, its effect on the LFM operation remains unknown [32,33]. Coincidentally, when there is a deviation angle between the force and the mode, it generates a component force in the orthogonal direction, which resembles the impact of the system phase error on the driving force deflection [34].

Indeed, it is noteworthy that all errors are inherently interactive, precluding absolute elimination and collectively impacting the SF and zero rate output (ZRO). Therefore, more precise compensation, control, and signal processing techniques are necessary. The focus of this paper is a fully symmetric resonator. Consequently, this paper investigates the interaction effects among stiffness coupling, system phase error, readout modulation phase shift, and velocity amplitude mismatch in the LFM gyroscope. The research then prioritizes the achievement of readout phase matching through the utilization of the fine impulse response (FIR) filter and the maintenance of frequency difference stability via in-phase control force as prerequisites. This thesis primarily addresses compensation for two specific errors. Specifically, the principle of electrostatic negative stiffness is employed to minimize stiffness coupling by applying an electrostatic voltage. Simultaneously, the system phase error is identified by observing the amplitude control force, and compensation is applied by adjusting the target value of the controller in the PPLs. Finally, the study concludes by demonstrating the impacts of these two errors on ZRO and the SF nonlinearity, accompanied by a detailed analysis of the underlying reasons.

## 2. Working Principle and Scheme Design

**Figure 2:**
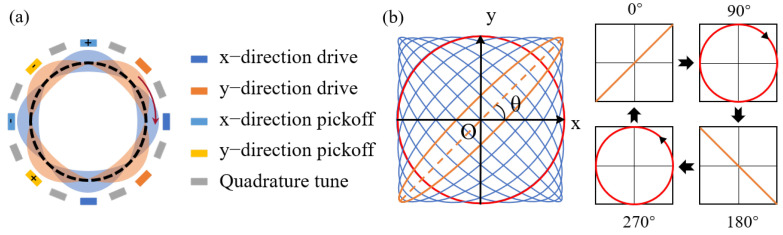
(**a**) n = 2 glass vibration mode with eight pairs of electrodes arranged around it, including one pair of driving electrodes and one pair of detecting electrodes on the X axis and Y axis and four pairs of electrodes used for quadrature correction. (**b**) With the continuous change in the relative phase between two axes, the vibration mode switches repeatedly from a standing wave to a traveling wave, and the displacement synthesis pattern changes continuously from a line to a circle, forming a standard Lissajous pattern.

The vibration mode of the fully symmetric gyroscope is typically depicted in Figure 2a as the n = 2 wineglass mode. In the LFM mode, the vibration exhibits a continuous transition between standing and traveling waves. An analysis of the trajectories derived from the changes in the X and Y axis displacements in Figure 2b reveals the alternating nature between lines and circles, resulting in the generation of a typical Lissajous pattern. Capacitive transformation can be achieved by sixteen distinct outer electrodes positioned outside the resonator’s lip. These out-of-plane electrodes can be used for excitation, detection, and electrostatic tuning. Furthermore, the symbols employed in the theoretical derivations and their corresponding descriptions are detailed in Table A1.

### 2.1. Dynamical Model of Gyroscope

**Figure 3:**
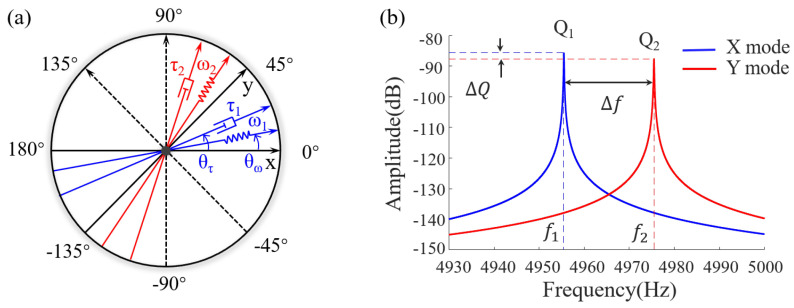
(**a**) Schematic diagram of the fully symmetric gyroscope model including the anisodamping error and frequency split. (**b**) The amplitude–frequency response of the principal mode in the LFM model, reflecting the quality factor and the resonant frequency of the resonator.

The dynamical characteristics of the fully symmetric MEMS gyroscope can be described as a mass–spring–damping system [35]. However, it should be noted that the physical period of the standing wave azimuth is half that of the lumped mass block. Taking the micro-hemisphere resonator as an example, as illustrated in Figure 3a, the model incorporates the errors arising from quality factor mismatch, frequency mismatch, damping axis deflection, and stiffness axis deflection between the principal mode X and the secondary mode Y. The dynamical model is described as follows:

(1) $$\begin{array}{cc} \left[\begin{array}{c} \ddot{x}\\ \ddot{y} \end{array}\right] & +\left(\left[\begin{array}{cc} d_{11} & d_{12}\\ d_{21} & d_{22} \end{array}\right]+4\alpha \Omega \left[\begin{array}{cc} 0 & -1\\ 1 & 0 \end{array}\right]\right)\left[\begin{array}{c} \dot{x}\\ \dot{y} \end{array}\right]+\left[\begin{array}{cc} k_{11} & k_{12}\\ k_{21} & k_{22} \end{array}\right]\left[\begin{array}{c} x\\ y \end{array}\right]=\left[\begin{array}{c} f_{x}\\ f_{y} \end{array}\right]. \end{array}$$

where *x* and *y* are the vibration displacements of the two modes, respectively. $f_{x}$ and $f_{y}$ are the control forces in the driving mode direction, respectively. α is the angular gain, and Ω is the input angular rate. The damping matrix is defined as

$$\left[\begin{array}{cc} d_{11} & d_{12}\\ d_{21} & d_{22} \end{array}\right]=\left[\begin{array}{cc} \frac{2}{\tau }+\Delta \left(\frac{1}{\tau }\right)\cos4\theta_{\tau } & \Delta \left(\frac{1}{\tau }\right)\sin4\theta_{\tau }\\ \Delta \left(\frac{1}{\tau }\right)\sin4\theta_{\tau } & \frac{2}{\tau }-\Delta \left(\frac{1}{\tau }\right)\cos4\theta_{\tau } \end{array}\right],$$

where 22τ τ represents the average damping of two modes, and the damping magnitude is the inverse of the time constant. Δ11τ τ represents the anisodamping. $\theta_{\tau }$ represents the azimuth of the principal damping axis, which is the angle between the maximum damping axis and the X axis. The stiffness matrix is expressed as

$$\left[\begin{array}{cc} k_{11} & k_{12}\\ k_{21} & k_{22} \end{array}\right]=\left[\begin{array}{cc} \omega^{2}-\omega \Delta \omega \cos4\theta_{\omega } & -\omega \Delta \omega \sin4\theta_{\omega }\\ -\omega \Delta \omega \sin4\theta_{\omega } & \omega^{2}+\omega \Delta \omega \cos4\theta_{\omega } \end{array}\right],$$

where $\omega^{2}$ represents the average stiffness of two modes, and the stiffness is squared with respect to the frequency. Δω represents the frequency split. $\theta_{\omega }$ represents the azimuth of the principal stiffness axis, which is the angle between the minimum stiffness axis and the X axis. Figure 3b shows the amplitude–frequency characteristics of the detected modes in the presence of the frequency split and anisodamping error.

### 2.2. Basic Working Principle of LFM

According to the averaging method, a sinusoidal approximation can be made for the X axis and Y axis displacement vibration signals as well as the drive signals. The displacements and control forces are assumed to be, respectively

(2) xy=Rexa·ei·ϕqxya·ei·ϕqy,

(3) fxfy=Refxc+ifxsei·ϕfxfyc+ifysei·ϕfy,

where *i* is the imaginary unit and Re· represents the operation of taking the real part. $x_{a}$ and $y_{a}$ represent the amplitudes of the X and Y mode displacement signals, respectively, while $\phi_{qx}$ and $\phi_{qy}$ represent the phases of the X and Y displacement signals, respectively. $f_{xc}$ and $f_{yc}$ denote the in-phase control force of the vibration, which can regulate the frequency. $f_{xs}$ and $f_{ys}$ denote the quadrature control force of the vibration, which can regulate the amplitude. $\phi_{fx}$ and $\phi_{fy}$ denote the phases of the X and Y mode drive signals, respectively. It should be noted that all of the aforementioned parameters are time-dependent functions.

The LFM operation necessitates equal vibration amplitudes for the two modes while maintaining a constant frequency difference. To fulfill the control condition, it is necessary to introduce a phase shift of ππ2 2 between $f_{xs}$ and $f_{ys}$ in relation to the phases of $x_{a}$ and $y_{a}$, respectively. This ensures the satisfaction of the condition $\phi_{qx}=\phi_{fx}=\phi_{x}$. Furthermore, the rate of change in the amplitude satisfies ${\dot{x}}_{a}={\dot{y}}_{a}\approx 0$. By incorporating the conditions and assumptions of (2) and (3) into (1), the phase method is employed to equate their real and imaginary parts. Finally, analytical expressions for the amplitudes and frequencies of the X and Y modes are derived:

(4) $$\;\omega_{x}=\left(4\alpha \Omega -d_{12}\right)\frac{v_{ya}}{v_{xa}}\sin\left(\Delta \phi_{xy}\right)+\frac{k_{11}}{\omega_{x}}+\frac{k_{12}}{\omega_{y}}\frac{v_{ya}}{v_{xa}}\cos\left(\Delta \phi_{xy}\right)-\frac{f_{xc}}{v_{xa}},$$

(5) $${\dot{v}}_{xa}=\left(4\alpha \Omega -d_{12}\right)\frac{v_{ya}}{2}\cos\left(\Delta \phi_{xy}\right)-\frac{d_{11}v_{xa}}{2}-\frac{k_{12}y_{a}}{2}\sin\left(\Delta \phi_{xy}\right)+\frac{f_{xs}}{2},$$

(6) $$\;\omega_{y}=\left(4\alpha \Omega +d_{21}\right)\frac{v_{xa}}{v_{ya}}\sin\left(\Delta \phi_{xy}\right)+\frac{k_{22}}{\omega_{y}}+\frac{k_{21}}{\omega_{x}}\frac{v_{xa}}{v_{ya}}\cos\left(\Delta \phi_{xy}\right)-\frac{f_{yc}}{v_{ya}},$$

(7) $$\;{\dot{v}}_{ya}=-\left(4\alpha \Omega +d_{21}\right)\frac{v_{xa}}{2}\cos\left(\Delta \phi_{xy}\right)-\frac{d_{22}v_{ya}}{2}+\frac{k_{21}x_{a}}{2}\sin\left(\Delta \phi_{xy}\right)+\frac{f_{ys}}{2}.$$

In the equation, $\omega_{x}$ and $\omega_{y}$ represent the instantaneous frequencies of the X and Y modes, respectively. $\Delta \phi_{xy}=\phi_{qy}-\phi_{qx}$ denotes the real-time phase difference between the two modes, while $v_{xa}=x_{a}\omega_{x}$ and $v_{ya}=y_{a}\omega_{y}$ correspond to the amplitude of the vibration velocity. (4)–(7) demonstrate the modulation effect of the frequency difference on the vibration displacement and reveal the evolution of manufacturing detects in the resonator in the LFM operation. The summation of (4) and (6) yields the following expression:

(8) $$\begin{array}{cc} \omega_{\Sigma xy}\;= & 4\alpha \Omega \left(\frac{v_{ya}}{v_{xa}}+\frac{v_{xa}}{v_{ya}}\right)\sin\left(\Delta \phi_{xy}\right)+\left(\frac{k_{22}}{\omega_{y}}+\frac{k_{11}}{\omega_{x}}\right)\\ & +d_{12}\left(\frac{v_{xa}}{v_{ya}}-\frac{v_{ya}}{v_{xa}}\right)\sin\left(\Delta \phi_{yx}\right)\\ & +\left(\frac{k_{21}}{\omega_{x}}\frac{v_{xa}}{v_{ya}}+\frac{k_{12}}{\omega_{y}}\frac{v_{ya}}{v_{xa}}\right)\cos\left(\Delta \phi_{xy}\right), \end{array}$$

where $\omega_{\Sigma xy}$ denotes the summation of two frequencies. After demodulation of this signal and low-pass filtering, an estimate of the angular rate can be obtained. The effectiveness of the LFM relies significantly on a robust control system, as it is through this system that all of the aforementioned control conditions are attained.

### 2.3. Control Scheme of the LFM

**Figure 4:**
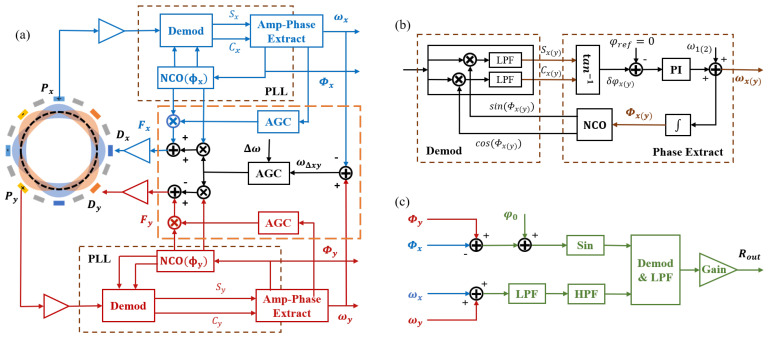
The basic scheme of the LFM operation. (**a**) The control scheme and X and Y modes are controlled independently, and the frequency tracking loop and amplitude control loop are set up, respectively. The modules added to the orange dashed box apply in-phase control forces to sustain the frequency difference. (**b**) The structure of the PLL is composed of a demodulator, a phase extractor, and a NCO, adopting a phase-sensitive demodulation method. (**c**) Structure of the readout module, applying the reference signal to demodulate the angular rate of the frequency.

The control scheme for the LFM mode is illustrated in Figure 4a, featuring separate control loops for the X and Y modes. Each loop comprises two components: amplitude control and frequency tracking. Taking the control structure of the X mode as an example, the vibration signal $P_{x}$ is extracted from the gyroscope and converted into a voltage signal using the C2V module. Subsequently, the voltage signal is demodulated with the reference signal from the previous moment to derive the slow variables $S_{x}$ and $C_{x}$. These two signals are combined through the amplitude and phase extraction module to obtain the current amplitude and phase. Finally, the amplitude control signal and the real-time phase are combined to generate the feedback control force $F_{x}$, which is then transformed into the drive voltage signal $D_{x}$ by the amplifier. The control structure for the Y mode follows a similar configuration. For clarity, the ADC and DAC modules are omitted from the figure.

Frequency difference control is necessary due to the frequencies of the X and Y modes shifting in opposite directions with an increase in the input angular rate. The in-phase control force of the vibration displacement is employed to adjust the resonant frequency without the need for additional components. The orange dashed box in Figure 4a illustrates the control of the instantaneous frequency difference $\omega_{\Delta xy}$ using AGC feedback to the frequency split. This control strategy generates control forces $f_{xc}$ and $f_{yc}$, which have equal magnitudes but opposite signs, enabling the adjustment of $\omega_{x}$ and $\omega_{y}$ in an inverse manner. Finally, the frequency and amplitude control forces combine to form the final drive signal as follows:

(9) $$\begin{array}{c} f_{x}=f_{xc}\cos\phi_{fx}-f_{xs}\sin\phi_{fx},\\ f_{y}=f_{yc}\cos\phi_{fy}-f_{ys}\sin\phi_{fy}. \end{array}$$

The form of (9) is consistent with the assumptions stated in (3). It is important to emphasize that the bandwidth of the controller should be significantly smaller than the frequency split value. This ensures that the frequency does not interfere with the modulation effect of the frequency by the angular rate.

The PLLs are established by the phase extractor and demodulator for tracking and reading instantaneous frequencies. The PLL structure of X modes is illustrated in Figure 4b, utilizing the phase-sensitive demodulation method. The slow variables are obtained as follows:

(10) $$\begin{array}{c} C_{x}=LPF\left\{A_{x}\sin\left(\phi_{qx}\right)\times 2\cos\left(\phi_{x}\right)\right\}=A_{x}\sin\left(\phi_{qx}-\phi_{fx}\right),\\ S_{x}=LPF\left\{A_{x}\sin\left(\phi_{qx}\right)\times 2\sin\left(\phi_{x}\right)\right\}=A_{x}\cos\left(\phi_{qx}-\phi_{fx}\right),\\ C_{y}=LPF\left\{A_{y}\cos\left(\phi_{qy}\right)\times 2\cos\left(\phi_{y}\right)\right\}=A_{y}\cos\left(\phi_{qy}-\phi_{fy}\right),\\ S_{y}=LPF\left\{A_{y}\cos\left(\phi_{qy}\right)\times 2\sin\left(\phi_{y}\right)\right\}=-A_{y}\sin\left(\phi_{qy}-\phi_{fy}\right). \end{array}$$

where $A_{x}$ and $A_{y}$ are the amplified amplitude signals. They have a constant target value in the controller, implying that the amplitude is sustained. The cut-off frequency of the low-pass filter (LPF) is set between the natural frequency and two times the natural frequency. Then, after the operation of the amp-phase extraction module, the expression of the slow-varying control quantity is obtained as (11).

(11) δφx=arctan(CxCxSx Sx)=ϕqx−ϕfx,Ax=Cx2+Sx2,δφy=−arctan(SySyCy Cy)=ϕqy−ϕfy,Ay=Cy2+Sy2.

where $\delta \varphi_{x}$ and $\delta \varphi_{y}$ denote the difference between the displacement vibration phase and the reference phase of the X and Y modes, respectively. The PI controller ensures precise frequency tracking by minimizing deviations to zero. It fine-tunes the natural frequency to obtain the instantaneous frequencies $\omega_{x}$ and $\omega_{y}$, which are then integrated to generate the instantaneous phases $\phi_{qx}$ and $\phi_{qy}$.

### 2.4. Readout Characteristics of the LFM

Figure 4c illustrates the process of the angular rate readout. $\omega_{\Sigma xy}$ is low-pass filtered to remove the high-frequency harmonic component. Then, high-pass filtering is performed to remove the constant component of the intrinsic frequency to obtain the modulated signal of the rate. The phases $\phi_{fx}$ and $\phi_{fy}$ generated by the PLLs are differenced, and the initial phase $\phi_{0}$ is added to obtain the phase of the demodulated reference signal. Finally, $sin\left(\Delta \phi_{xy}\right)$ is generated for in-phase demodulation, and the ideal readout of the LFM is obtained after low-pass filtering again, as follows:

(12) $$R_{out}=2\alpha v_{rrs}\times \Omega +d_{12}v_{rrd}.$$

where vrrs=vyavyavxa vxa+vxavxavya vya denote the reciprocal sum of the vibration velocity ratio, and vrrd=vxavxavya vya−vyavxavxavya vya−vyavxa vxa denote the reciprocal difference. When the velocity amplitude mismatch is small enough, $v_{rrs}\approx 2$ and $v_{rrd}\approx 0$. Meanwhile, the readout of the sense mode of the AM gyroscope excluding the quadrature error is [36]:

(13) $$Y_{out}=\frac{A_{x}}{\sqrt{{(2\Delta \omega )}^{2}+{\left(\omega /Q_{2}\right)}^{2}}}\left(4\alpha \times \Omega +d_{12}\right).$$

Unlike the intricate scale factor associated with the AM mode, the output of the LFM mode (12) does not contain the resonant frequency and quality factor, the primary contributors to temperature sensitivity. Although the $\omega_{\Sigma xy}$ in the LFM exhibits slow variations with the ambient temperature, these changes are significantly smaller than the split frequency. The high-pass filter effectively mitigates this variation, resulting in excellent temperature stability for the LFM. Furthermore, the LFM mode demonstrates an immunity to the anisodamping error when compared to the AM mode due to $v_{rrd}\approx 0$. However, these advantages are accompanied by the limitation of the bandwidth in the LFM.

Thanks to the accurate frequency detection, the phase of the demodulated reference signal can be obtained directly as $\Delta \phi_{xy}=\int \left(\omega_{y}-\omega_{x}\right)dt$. The integration operation introduces a phase lag of ππ2 2, which requires overcompensation for the demodulated signal. Nevertheless, the filtered signal $\omega_{\Sigma xy}$ introduces a phase delay with respect to $sin\left(\Delta \phi_{xy}\right)$, and this delay is also subject to variations in $\omega_{\Sigma xy}$. Therefore, real-time phase matching becomes necessary. One potential solution to this problem is the utilization of the class-I FIR filter, which is characterized by a group delay that is expressed as follows in the frequency domain:

(14) $$\tau_{g}\left(\omega \right)=\frac{d\theta \left(\omega \right)}{d\omega }=-\frac{N-1}{2}.$$

In digital signal processing, the unit of group delay is typically measured in samples, and the corresponding delay can be calculated based on the sampling frequency. The linear phase characteristic of the FIR filter ensures that real-time phase matching can be achieved by simply delaying $sin\left(\Delta \phi_{xy}\right)$ by a fixed number of samples. This approach remains effective, regardless of how the real-time frequency difference may change.

## 3. Interactive Error Analysis and Correction

The stiffness coupling and velocity amplitude mismatch were previously addressed in the section where the LFM dynamic equations were established. To examine the interaction effect among these errors, this section takes into account the impact of both the system phase error and the readout module phase shift. The resulting LFM output expression incorporates all relevant errors. Subsequent discussion is centered on the identification and compensation methods for stiffness coupling and system phase error, respectively.

### 3.1. Analysis of Interaction Effect

In practical control systems, phase errors inevitably exist, causing a phase lag between the control signal and the displacement. Due to the consistent structure, the phase error of the X mode is almost equal to that of the Y mode. Therefore, it is reasonable to assume that

(15) $$\phi_{qx}-\phi_{fx}=\phi_{qy}-\phi_{fy}=\delta \varphi ,$$

where δφ represents the phase error. With this assumption, the expressions for (4) and (6) are updated as follows:

(16) $$\begin{array}{c} \;\omega_{x}=\left(4\alpha \Omega -d_{12}\right)\frac{v_{ya}}{v_{xa}}\frac{\sin\left(\Delta \phi_{xy}+\delta \varphi \right)}{\cos\left(\delta \varphi \right)}+\frac{k_{11}}{\omega_{x}}+\frac{k_{12}}{\omega_{y}}\frac{v_{ya}}{v_{xa}}\frac{\cos\left(\Delta \phi_{xy}+\delta \varphi \right)}{\cos\left(\delta \varphi \right)}-d_{11}\tan\left(\delta \varphi \right), \end{array}$$

(17) $$\begin{array}{c} \;\omega_{y}=\left(4\alpha \Omega +d_{21}\right)\frac{v_{xa}}{v_{ya}}\frac{\sin\left(\Delta \phi_{xy}-\delta \varphi \right)}{\cos\left(\delta \varphi \right)}+\frac{k_{22}}{\omega_{y}}+\frac{k_{21}}{\omega_{x}}\frac{v_{xa}}{v_{ya}}\frac{\cos\left(\Delta \phi_{xy}-\delta \varphi \right)}{\cos\left(\delta \varphi \right)}-d_{22}\tan\left(\delta \varphi \right). \end{array}$$

It can be observed that the phase error affects the modulation effect of the rate on the frequency and directly influences the frequency offset through damping. Meanwhile, there is a phase shift Δφ between $\omega_{\Sigma xy}$ and $sin\left(\Delta \phi_{xy}\right)$ caused by the filter. Thus, (12) is updated as follows:

(18) $$\begin{array}{ll} R_{out}\;= & 2\alpha \left(v_{rrs}\cos\left(\Delta \varphi \right)+\underset{Item1}{\underset{︸}{v_{rrd}\tan\left(\delta \varphi \right)\sin\left(\Delta \varphi \right)}}\right)\times \Omega +\underset{Item2}{\underset{︸}{d_{12}v_{rrd}\cos\left(\Delta \varphi \right)}}+\underset{Item3}{\underset{︸}{d_{12}v_{rrs}\tan\left(\delta \varphi \right)\sin\left(\Delta \varphi \right)}}\\ & -\underset{Item4}{\underset{︸}{\left(\frac{k_{21}}{\omega_{x}}\frac{v_{xa}}{v_{ya}}-\frac{k_{12}}{\omega_{y}}\frac{v_{ya}}{v_{xa}}\right)\tan\left(\delta \varphi \right)\cos\left(\Delta \varphi \right)}}-\underset{Item5}{\underset{︸}{\left(\frac{k_{21}}{\omega_{x}}\frac{v_{xa}}{v_{ya}}+\frac{k_{12}}{\omega_{y}}\frac{v_{ya}}{v_{xa}}\right)\sin\left(\Delta \varphi \right)}} \end{array}$$

This indicates that stiffness coupling, the anisodamping error, the system phase error, the phase shift in the readout module, and velocity amplitude mismatch collectively influence the ZRO and SF of the gyroscope, resulting in complex interaction effects. In theory, individually eliminating each error can significantly optimize the performance. However, each of these influences cannot be completely removed. Therefore, while frequency difference control and FIR filters have been employed to mitigate the velocity mismatch and phase shift, it remains imperative to suppress stiffness coupling and compensate for the phase error to optimize the output performance.

### 3.2. Suppression of Stiffness Coupling by Quadrature Voltage

**Figure 5:**
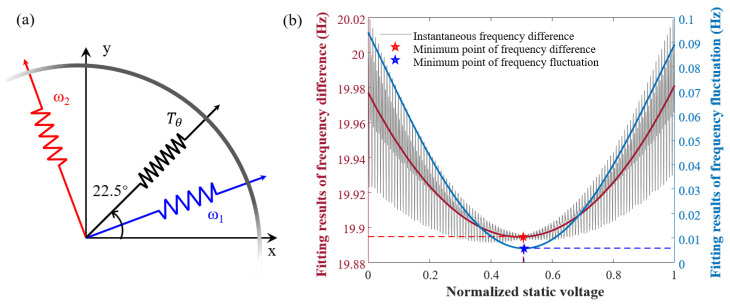
(**a**) Applying a stiffness load at the 22.5° position alters the stiffness distribution of the resonator. (**b**) The bias and fluctuations (peaks and valleys) in the frequency difference change as the equivalent stiffness load of the quadrature voltage varies. The minimum point is reached when they are equal.

Stiffness coupling is caused by both Δω and $\theta_{\omega }$. The LFM gyroscope intentionally maintains a large frequency split that cannot be altered. In fully symmetric resonators, the azimuth of the principal stiffness axis is typically substantial. Its size depends on the accuracy of identifying the stiffness axis and the relative electrode installation since it lacks physical constraints. Therefore, stiffness coupling has to be suppressed by adjusting the angle using quadrature electrodes located at 22.5°, as shown in Figure 5a. By applying a static DC voltage to the resonator, based on the electrostatic negative stiffness effect, the voltage can be equivalently regarded as a stiffness load that alters the degree of coupling between the stiffness in the X and Y modes. Under this influence, the stiffness matrix is represented as follows:

$$\left[\begin{array}{cc} k_{11} & k_{12}\\ k_{21} & k_{22} \end{array}\right]=\left[\begin{array}{cc} \omega^{2}-\omega \Delta \omega \cos4\theta_{\omega }+T_{\theta } & -\omega \Delta \omega \sin4\theta_{\omega }-T_{\theta }\\ -\omega \Delta \omega \sin4\theta_{\omega }-T_{\theta } & \omega^{2}+\omega \Delta \omega \cos4\theta_{\omega }+T_{\theta } \end{array}\right],$$

where $T_{\theta }$ is the stiffness load, which is negatively proportional to the electrostatic voltage. The purpose of stiffness coupling suppression is to make $T_{\theta }=-\omega \Delta \omega \sin4\theta_{\omega }$ by tuning the electrostatic voltage. The resonant frequencies of the X and Y modes can be calculated from the characteristic values of the stiffness matrix. When $T_{\theta }=0$, $\omega_{x}=\omega_{1}$ and $\omega_{y}=\omega_{2}$ are calculated, indicating that the instantaneous frequencies can be tracked to the natural frequencies under normal circumstances. As $\omega_{x}$ and $\omega_{y}$ vary with the adjustment of $T_{\theta }$, it results in a continuous decrease in the instantaneous frequency difference $\omega_{\Delta xy}$, where $\omega_{\Delta xy}$ = $\omega_{y}-\omega_{x}$. Additionally, the fluctuation in the frequency difference varies with the stiffness coupling. Figure 5b illustrates a least squares fit to both the instantaneous frequency difference and the frequency fluctuation.

**Figure 6:**
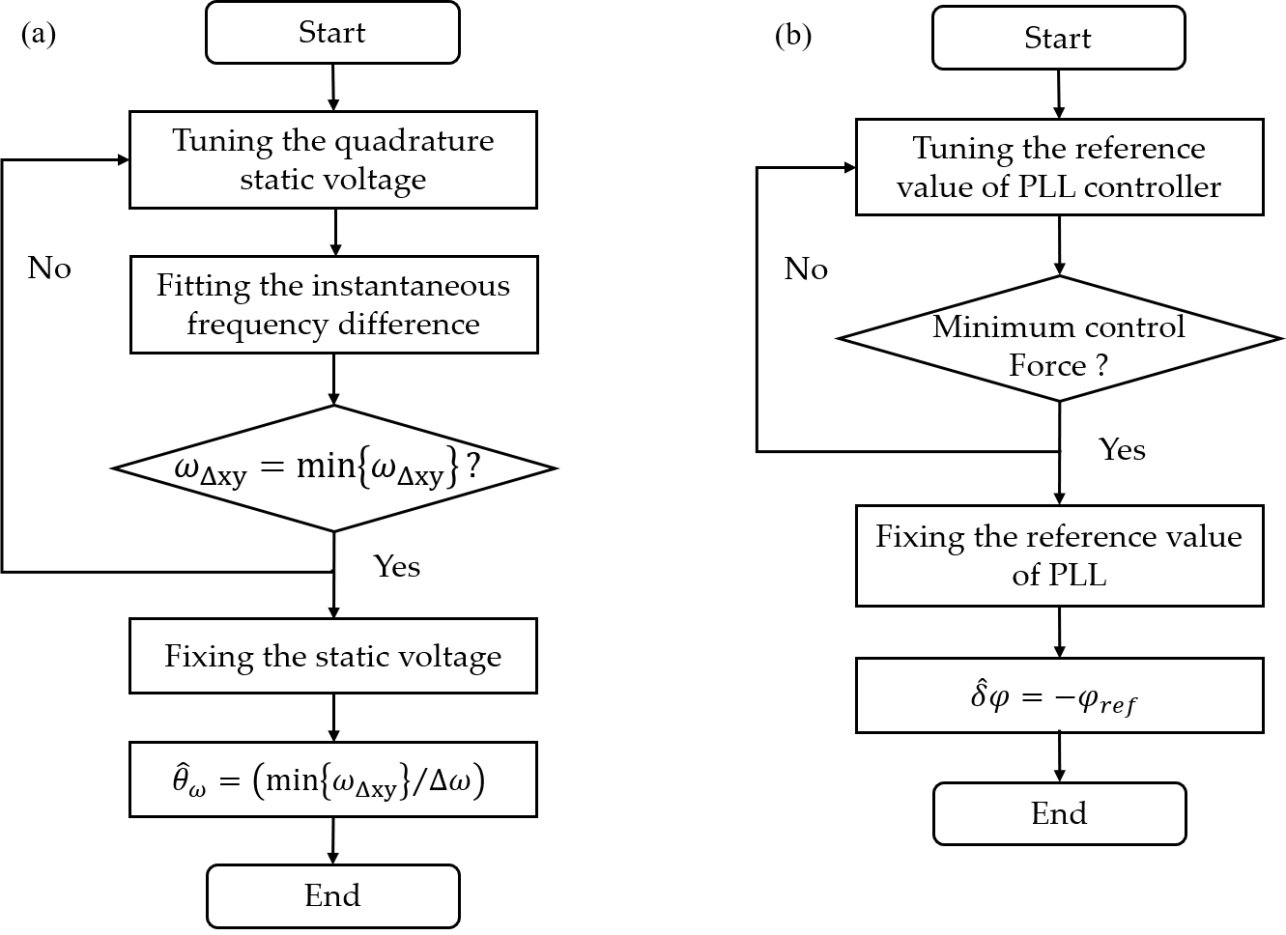
(**a**) The flowchart for stiffness coupling identification and suppression. (**b**) The flowchart for system phase error identification and compensation.

When $T_{\theta }=-\omega \Delta \omega \sin4\theta_{\omega }$, $\omega_{\Delta xy}$ reaches the minimum value. Hence, the flowchart for stiffness coupling suppression is illustrated in Figure 6a. The value of $\omega_{\Delta xy}$ can be directly read, and its minimum can be observed by continuously adjusting the quadrature voltage. Once the voltage at the minimum frequency difference is determined, the stiffness coupling can be eliminated by applying a constant voltage. Additionally, the minimum points for both of them correspond to approximately the same electrostatic voltage level. Furthermore, the value of $\theta_{\omega }$ can be estimated as follows:

(19) $${\hat{\theta }}_{\omega }=\arccos\left(\frac{\min\left\{\omega_{\Delta xy}\right\}}{\Delta \omega }\right).$$

where $\min\left\{\cdot \right\}$ represents the operation of obtaining the minimum value.

### 3.3. Identification and Compensation for the System Phase Error

**Figure 7:**
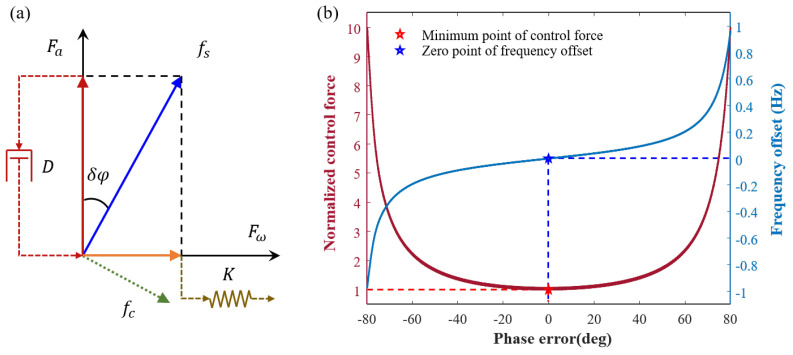
(**a**) Due to phase error, the direction of the quadrature control force $f_{s}$ experiences a deviation. To ensure that the amplitude control force $F_{a}$ effectively counteracts the damping, $f_{s}$ will increase. Additionally, this leads to the generation of an orthogonal component $F_{w}$ which, in turn, changes the resonant frequency of the mode. (**b**) As the phase error of the system continuously varies from 80° lagged to 80° ahead, the quadrature control force (red) initially decreases, then increases, and reaches its minimum at the zero point. Simultaneously, the resonant frequency (blue) decreases, then increases, and reaches the natural frequency at the zero point.

The system phase error is caused by different components of the circuit, including voltage-capacitor drive, resonator, capacitor-voltage conversion, and slow variable demodulation. Figure 7a illustrates that the phase error of the system can be attributed to the phase lag of the quadrature control force $f_{s}$. When the amplitude control loop is stable without input angular rate, the quadrature control signal can be expressed as follows:

(20) $$F_{xa}=\left|f_{xs}\right|\cos\left(\delta \varphi \right)=d_{11}v_{xa}-d_{12}\frac{\omega_{y}\cos\left(\Delta \phi_{xy}+\delta \varphi \right)}{\cos\left(\delta \varphi \right)},$$

(21) $$F_{ya}=\left|f_{ys}\right|\cos\left(\delta \varphi \right)=d_{22}v_{ya}+d_{21}\frac{\omega_{x}\cos\left(\Delta \phi_{xy}-\delta \varphi \right)}{\cos\left(\delta \varphi \right)},$$

where $F_{xa}$ and $F_{ya}$ represent the amplitude control forces for the X and Y modes, respectively. $\left|\cdot \right|$ denotes the mode selection. Taking the X mode as an example, to balance the damping with $F_{xa}$, the magnitude of $f_{xs}$ varies with δφ. $f_{xs}$ is minimized when δφ=0, and $F_{xa}=f_{xs}$.

As depicted by the red curve in Figure 7b, the quadrature control force exhibits a cosecant relationship as the system phase error is continuously adjusted from −80° to 80°. The control force is minimized when the phase error is zero. Additionally, the resonant frequency offset also varies with the phase error, as indicated by the blue curve in Figure 7b, showing a tangential relationship between them. The zero point of the phase error corresponds to the zero point of the frequency offset.

Therefore, the variation in the control force can be monitored by continuously adjusting its phase. When the control force reaches a minimum value, it signifies the cancellation of the phase error. Similarly, the phase can be continuously adjusted to align the frequency with the natural frequency and match the system phase error value. However this method is less stable due to the susceptibility of the stiffness to temperature fluctuations.

**Figure 8:**
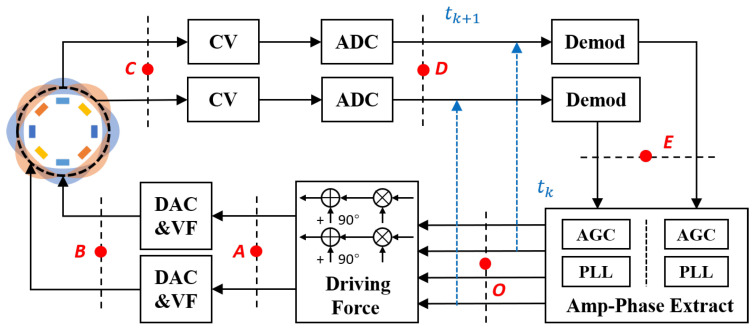
The phase error signal flow can be represented by a block diagram, where the initial phase is the output of the PLL in Amp-Phase Extract block. As the signal flows through each block in the system, it accumulates a phase shift.

Nevertheless, the actual circuit lacks an interface for additional adjustment of the control force phase. Therefore, it becomes imperative to identify an adjustable parameter as an alternative. Figure 8 illustrates the transmission of the phase error within the loop. When using the output of the PLL as the initial phase, the signal at point *O* remains unshifted in phase, while each subsequent block undergoes a phase lag. The 90° phase shift occurs after driving force synthesis. The phase lags generated by the voltage–capacitor drive, resonator, capacitor–voltage conversion, and slow variable demodulation are denoted as $\phi_{1}$, δϕ, $\phi_{2}$, and $\phi_{3}$, respectively. Table 1 illustrates the signal forms corresponding to nodes *A*, *B*, *C*, *D*, and *E*. Subsequently, the system phase error can be expressed as

(22) $$\delta \varphi =90^{\circ }+\delta \phi +\phi_{1}+\phi_{2}+\phi_{3}.\;$$

At time $t_{k}$, the signal at point *D* is demodulated by the signal at point *O*. Subsequently, the in-phase and quadrature signals at point *E* are generated, exhibiting a phase lag. The phase difference is then determined from these signals, and the reference phase at time $t_{k+1}$ is obtained using the zero-error controller of the PLLs. Due to ππ2 2+δϕ=0, a static error caused by (22) is consistently present in the output of the PLLs, as shown in Figure 4b. In other words, the displacement phase $\phi_{q}$ remains unsynchronized with the force phase $\phi_{f}$.

The crucial aspect is that the reference value $\varphi_{ref}$ within the PLL controller serves as a modifiable parameter interface. Thus, phase errors can be identified by tuning $\varphi_{ref}$ instead of adjusting the control phase. Specifically, instead of setting $\varphi_{ref}$ to zero, it is set to −δφ when extracting the phase difference from point *E*. This corresponds to overcompensation of the lag within the loop. The flowchart for system phase error compensation is illustrated in Figure 6b. In this case, point *D* is always completely synchronized with the reference signal, which means that the phase error is compensated for. And, the phase error can be obtained by $\hat{\delta }\varphi =-\varphi_{ref}$.

## 4. Validations and Discussion

**Figure 9:**
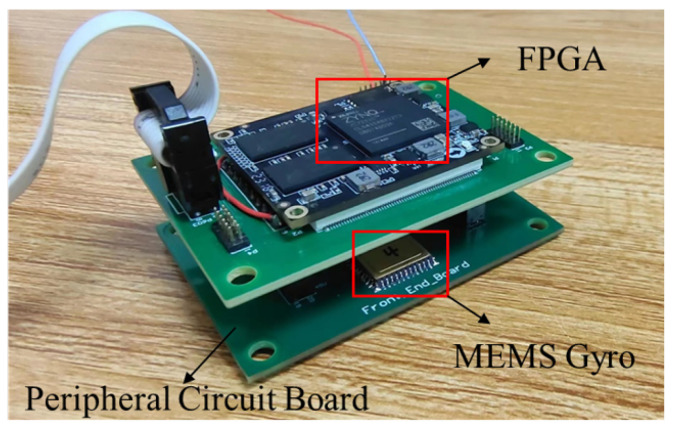
MEMS gyroscope digital verification platform, including the MEMS gyroscope, FPGA, and peripheral circuits.

Experiments are designed for comparison and validation to verify the effectiveness of the error discrimination-based compensation and optimal control strategy proposed in this paper for the performance improvement of the LFM gyroscope with the interaction effect. We designed and implemented a LFM test board as shown in Figure 9. The gyroscopes of the same batch have been tested to obtain the following approximate parameters, as shown in Table 2, including the resonator characteristics and the system error.

**Figure 10:**
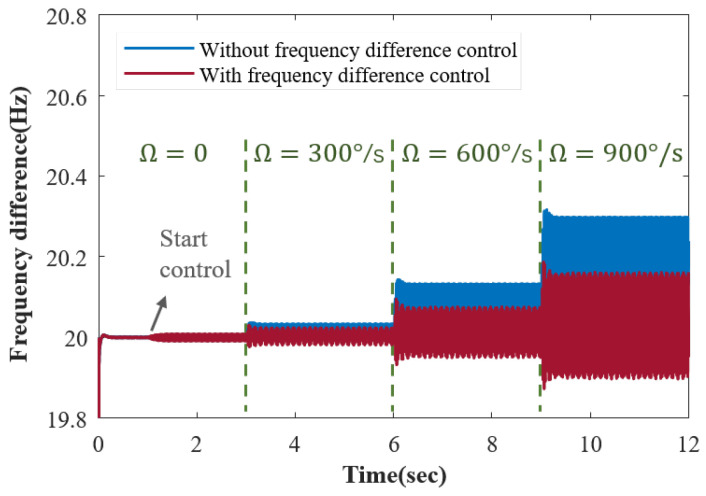
The input angular rate has a notable influence on the frequency difference. The controlled frequency difference (red curve) exhibits a reduced offset compared to the uncontrolled result (blue curve), leading to an improvement in sustaining the desired frequency difference. The control action is initiated at the 1 s mark.

Across a wide measurement range, the velocity amplitude mismatch effect becomes more pronounced, amplifying the adverse consequences produced from error interaction. As depicted by the blue curve in Figure 10, when the input angular rate is set at stages of 0, 300°/s, 600°/s, and 900°/s, the frequency difference offset exhibits a stepwise increase without frequency difference control. As illustrated by the red curve, in-phase force frequency difference control constrains the increment in the frequency difference offset with the increasing angular rate. This effect is achieved without resorting to electrostatic voltage control.

**Figure 11:**
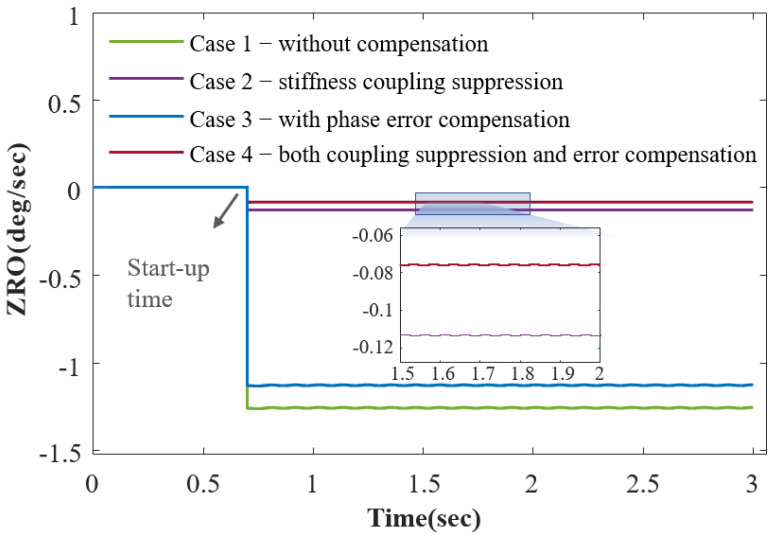
The ZRO results are compared for four cases: Case 1, without stiffness coupling suppression and phase error compensation; Case 2, with stiffness coupling suppression; Case 3, with phase error compensation; and Case 4, with both suppression and compensation. The enlarged graph highlights the optimal outcome achieved by simultaneously eliminating both stiffness coupling and the phase error.

Equation (18) indicates that factors such as stiffness coupling, anisodamping error, system phase error, demodulation phase shift, and velocity amplitude mismatch contribute to ZRO and SF. The ZRO of the LFM operation reflects the gyroscope’s zero bias level. With frequency difference control and demodulation phase matching as the foundation, Items 1 to 5 of (18) are significantly mitigated. Nevertheless, stiffness coupling and phase error can amplify the residuals in Item 1, Item 3, and Item 5. Figure 10 illustrates a comparison of the results obtained from four sets of experiments, where each case examines the ZRO from start-up to output stabilization over a 3 s duration. The results are smoothed to aid in the visual comparison of the bias. The results indicate that the individual suppression of stiffness coupling can significantly reduce the ZRO. Compensating for the system phase error can further reduce the ZRO, although the reduction is not substantial. On one hand, (18) reveals that the phase error is in the form of a tangent, and the tangent of a few degrees is quite small. On the other hand, when stiffness coupling is mitigated, the phase error appears in the form of a product with the anisodamping error in Item 3, further constraining their impact. Moreover, Figure 11 presents a stable start-up time of less than 0.7 s.

Furthermore, despite the general belief that anisodamping in LFM mode does not interfere with the output, the simulation results confirm the existence of the anisodamping error’s impact, albeit small. Item 2 and Item 3 of (18) reveal its presence in the form of interactions with other errors. Previous research has oversimplified the theory, overlooking these subtle effects, thereby constraining further enhancements in the LFM gyroscope accuracy. Naturally, the quality factor of a symmetrical structure resonator can be intentionally designed to be exceptionally high, thereby effectively mitigating this effect.

**Figure 12:**
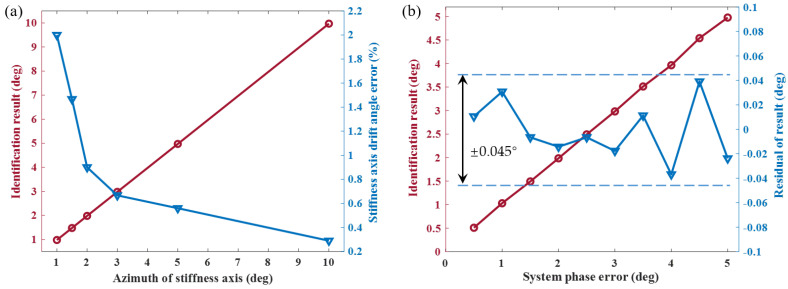
(**a**) The identification results of the azimuth of the principal stiffness axis and the errors of identification. There is an approximate 0.03° error present in each discrimination. (**b**) The identification results of the system phase error with a residual error of approximately 0.045°.

In the process of suppressing stiffness coupling, the azimuth of the principal stiffness axis can be estimated using (19). As illustrated in Figure 12a, the discrimination results for azimuths of 1°, 1.5°, 2°, 3°, 5°, and 10° are obtained through numerical simulation. There is a notable agreement between the set values and the discrimination results, with the estimation error decreasing as the azimuth increases. This phenomenon can be attributed to the approximate 0.03° error present in each discrimination. The magnitude of this error is dependent on the accuracy of frequency detection and the resolution of the voltage that can be applied. Likewise, Figure 12b illustrates the outcomes of detecting 10 phase errors ranging from 0.5 degrees to 5 degrees at intervals of 0.5 degrees with an identification error of approximately 0.045°. This is attributed to the negligible variation in the control force as the phase error approaches zero, limiting the improvement in the identification accuracy.

**Figure 13:**
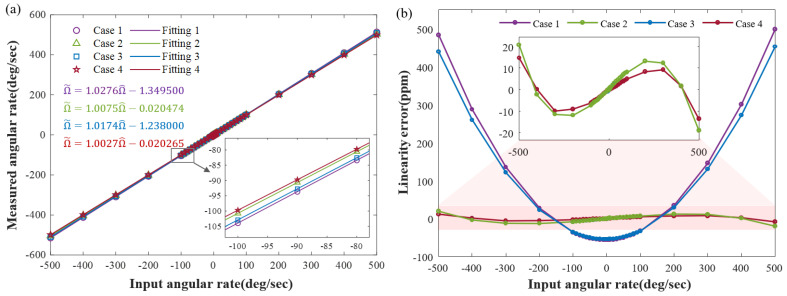
(**a**) The solid line represents the least squares fit of data in each case, and the results are color-coded. Additionally, the changes in the bias and SF were magnified. The best accurate output is obtained by suppressing both the stiffness coupling and phase error. (**b**) The linearity error of the SF is evaluated for the four cases within the measurement range of ±500°/s, and a magnified comparison between Case 2 and Case 4 is provided. Suppression of stiffness coupling drastically reduces the SF nonlinearity, and compensation of phase error further optimizes the results.

To elucidate the factors influencing SF nonlinearity, we conducted simulations to assess the output performance in four cases set earlier. Figure 13a illustrates the results of different angular rate inputs and outputs for them, ranging from −500°/s to 500°/s. The data were fitted using the least squares method, and the results are color-coded in the figure. These fitting results can be further employed to correct the output SF and bias. The comparison between Case 1 and Case 4 reveals that both stiffness coupling and phase error notably impact the SF and the constant bias. Moreover, the comparison between Case 2 and Case 3 reveals that the impact of stiffness coupling on bias is more pronounced than that of phase error. Simultaneously addressing both aspects can optimize the accuracy of the output.

Both the variation in the SF and bias with the angular rate can lead to the nonlinearity of the full measurement SF. The output exhibits rate dependence, which can be quantified by the SF linearity error. To illustrate the impacts of different factors on the SF nonlinearity, we conducted a comparison of linearity errors in four cases as well. Figure 13b illustrates that the results of Case 2 exhibited a noteworthy improvement in comparison to Case 1. This change can be primarily attributed to the substantial magnitude of stiffness coupling in Item 5 of (18), coupled with the residual demodulation phase shift, leading to zero bias disturbances. It is worth noting that the curve of Case 1 exhibits even symmetry and that of Case 2 displays odd symmetry. This is due to the fixed sign of Item 5, whereas the sign for Item 3 varies in accordance with the sign angular rate. The results of Case 3 compared to Case 1 and Case 4 compared to Case 2 are both flatter and have the same trend. This indicates that a slight phase error can also contribute to nonlinearity. The maximum linearity error is defined to represent the SF nonlinearity of the gyroscope. Within the measurement range of ±500°/s, the SF nonlinearity for Case 1 and Case 4 is 500 ppm and 15 ppm, respectively. These results suggest that the interaction effect analysis and error correction can enhance the gyroscope’s linearity by approximately 33 times.

**Figure 14:**
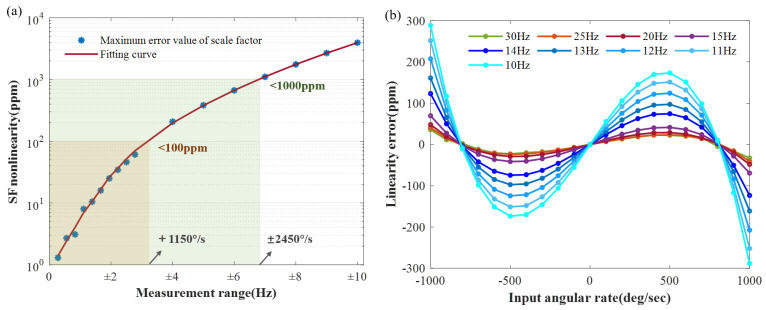
(**a**) The numerical simulation results demonstrate the SF nonlinearity for various measurement ranges. The orange area represents a measurement range of ±1150°/s, which corresponds to a nonlinearity below 100 ppm. Similarly, the green area represents a measurement range of ±2450°/s, corresponding to a nonlinearity of below 1000 ppm. (**b**) The comparison results for the scale factor linearity errors at different frequency splits within a measurement range of ±1000°/s. The results indicate that, as the frequency split approaches the measurement range, its effect on limiting the linearity becomes more pronounced. Conversely, when the frequency split is sufficiently distant from the measurement range, this limitation dissipates.

Due to the correlation between the results of nonlinearity and the measurement range, numerical simulations were conducted for different measurement ranges. A total of eighteen measurement ranges were selected, ranging from ±100°/s to ±1000°/s with an interval of 100°/s and from ±1440°/s (±4Hz) to ±3600°/s (±10Hz) with an interval of 360°/s. As illustrated in Figure 14a, after obtaining the SF nonlinearity within the corresponding measurement range, fitting is conducted. Numerically, nonlinearity at 100 ppm corresponds to ±1150°/s, meeting high precision requirements, while 1000 ppm corresponds to ±2450°/s, meeting consumer-grade stability requirements. The maximum tested measurement range is approximately ±10 Hz, corresponding to a 10 Hz bandwidth. From a trend perspective, as the measurement range increases, the SF nonlinearity rapidly rises. This can be attributed to the rapid decline in the amplitude control and the frequency difference control capabilities at large rates, leading to a significant increase in the velocity amplitude mismatch. Additionally, as the angular rates approach the bandwidth, they may cause disturbances akin to modulation frequencies in the system, resulting in a rapid performance degradation.

To confirm this conjecture, the nonlinearity of the SF was tested at a range of 1000°/s across different frequency splits, ranging from 10 Hz to 30 Hz. The results, displayed in Figure 14b, depict the linearity errors for nine distinct frequency splits, specifically 30 Hz, 25 Hz, 20 Hz, and 15 Hz to 10 Hz. Each group comprises 21 data points, collected in the range from −1000°/s to 1000°/s, with increments of 100°/s. For each set of simulations, the parameters of the filters in the calculation module were adjusted accordingly, forming the foundation for normal system operation. The simulation results suggest that, within a certain range, scale factor nonlinearity is correlated with the frequency split setting. Larger splits result in less nonlinearity, but there is a limit beyond which increasing the split does not further reduce nonlinearity. Since the control parameters remained constant, controller-related factors were ruled out. Therefore, this phenomenon may be attributed to rates deviating from the range of system modulation frequency perturbations. This observation can assist with the selection of LFM’s frequency split.

## 5. Conclusions

To address the limitation of SF nonlinearity in wide measurement ranges of LFM fully symmetric MEMS gyroscopes, this paper investigates the impact of the interaction between three typical errors and the velocity amplitude mismatch. Subsequently, a step-by-step error compensation and control optimization scheme is proposed to mitigate each contributing factor and achieve a noteworthy reduction in the SF nonlinearity. Initially, a dynamic model was established, encompassing stiffness coupling, system phase error, readout phase shift, and velocity mismatch. Meanwhile, we analyzed the mutual interactions among these factors, along with their impacts on the output. Next, in-phase force frequency difference control and FIR filter phase matching were implemented as basic conditions, mitigating the velocity mismatch and demodulation phase shift, respectively. Finally, we eliminated stiffness coupling and calibrated the phase error using the quadrature voltage and modification of the PLL reference, respectively. Compared to the original scheme, the SF nonlinearity decreased from 500 ppm to 15 ppm within the measurement range of ±500°/s, representing a 97% reduction. Furthermore, it was discovered that ensuring a sufficiently large frequency split effectively suppresses the SF nonlinearity. The simulation results closely align with the theoretical analysis, offering valuable guidance for enhancing the SF stability in LFM MEMS gyroscopes, particularly in the context of manufacturing process limitations.

## Figures and Tables

**Table 1:** The phase of the signal flow at different points of the system.

Signal Flow Point	Signal Flow Phase
*O*	$\cos\left(\phi \right)$
*A*	$\cos\left(\phi +90^{\circ }\right)$
*B*	$\cos\left(\phi +90^{\circ }+\phi_{1}\right)$
*C*	$\cos\left(\phi +90^{\circ }+\phi_{1}+\delta \phi \right)$
*D*	$\cos\left(\phi +90^{\circ }+\phi_{1}+\delta \phi +\phi_{2}\right)$
*E* ^1^	$\begin{array}{c} \cos\left(\phi +90^{\circ }+\phi_{1}+\delta \phi +\phi_{2}+\phi_{3}\right)\\ -\sin\left(\phi +90^{\circ }+\phi_{1}+\delta \phi +\phi_{2}+\phi_{3}\right) \end{array}$

^1^ The phase difference between the demodulated signal of the in-phase and quadrature directions is π/2.

**Table 2:** Key parameters used in the simulation.

Symbol	Description	Value	Unit
$\omega_{1}$	primary modal frequency	4955.5 × 2π	rad
$\omega_{2}$	secondary modal frequency	4975.5 × 2π	rad
Δω	initial frequency split	20 × 2π	rad
$Q_{1}$	primary modal quality factor	50,000	
$Q_{2}$	secondary modal quality factor	48,000	
$\theta_{\omega }$	azimuth of principal stiffness axis	1.5	deg
$\theta_{\tau }$	azimuth of principal damping axis	5	deg
δϕ	system phase error	5	deg
δφ	initial demodulation phase shift	π/3	rad

## Data Availability

Data are contained within the article.

## Abbreviations

The following abbreviations are used in this manuscript:

MEMS	Micro-Electro-Mechanical System
SF	Scale Factor
ZRO	Zero Rate Output
AM	Amplitude Modulated
FM	Frequency Modulated
QFM	Quadrature Frequency Modulated
IFM	Indexed Frequency Modulated
LFM	Lissajous Frequency Modulated
ASIC	Application-Specific Integrated Circuit
PLL	Phase-Locked Loop
AGC	Automatic Gain Control
CSWaP	Cost, Size, Weight, and Power
NCO	Numerically Controlled Oscillator
FIR	Finite Impulse Response
ADC	Analog-to-Digital Converter
DAC	Digital-to-Analog Converter
PI	Proportional Integral

## Appendix A. Glossary

The list of symbols used in this manuscript. The key parameters in Table 2 are not repeated here.

**Table A1:** Symbols used in this manuscript.

Symbol	Description	Symbol	Description
x,y	vibration displacements of the X and Y modes	α	angular gain
$f_{x},f_{y}$	control forces of the X and Y modes	Ω	input angular rate
$x_{a},y_{a}$	amplitudes of the vibration displacements	$d_{11},d_{22}$	damping of the X and Y modes
${\dot{x}}_{a},{\dot{y}}_{a}$	change rates of the amplitudes	$d_{12},d_{21}$	damping coupling between the X and Y modes
$\phi_{qx},\phi_{qy}$	phases of the vibration displacements	$k_{11},k_{22}$	stiffness of the X and Y modes
$\phi_{fx},\phi_{fy}$	phases of the control forces	$k_{12},k_{21}$	stiffness coupling between the X and Y modes
$f_{xc},f_{yc}$	in-phase control forces of the X and Y modes	2/τ	average damping between the X and Y modes
$f_{xs},f_{ys}$	quadrature control forces of the X and Y modes	Δ(1/τ)	anisodamping between the X and Y modes
$F_{xa},F_{ya}$	amplitude control forces of the X and Y modes	$\omega^{2}$	the average stiffness between the X and Y modes
$\omega_{x},\omega_{y}$	the instantaneous frequencies of the X and Y modes	$\Delta \phi_{xy}$	real-time phase difference in the displacements
$v_{xa},v_{ya}$	amplitudes of the vibration velocity	$v_{rrs}$	the reciprocal sum of the velocity ratio
${\dot{v}}_{xa},{\dot{v}}_{ya}$	change rates of the vibration velocity amplitudes	$v_{rrd}$	the reciprocal difference of the velocity ratio
$\delta \varphi_{x},\delta \varphi_{y}$	difference between the displacement phase and the force phase of the X and Y modes	$\omega_{\Sigma xy}$	summation of the instantaneous frequencies
$A_{x},A_{y}$	amplified amplitudes of the displacements	$\omega_{\Delta xy}$	difference in the instantaneous frequencies
$\phi_{1},\phi_{2},\phi_{3}$	phase lags generated by blocks in the loop	δϕ	phase lag generated by the resonator
